# Diabetic Ketoacidosis With Refractory Hypokalemia Leading to Cardiac Arrest

**DOI:** 10.7759/cureus.23439

**Published:** 2022-03-24

**Authors:** Sarah Grout, Danielle Maue, Zachary Berrens, Nathan Swinger, Stefan Malin

**Affiliations:** 1 Department of Pediatrics, Indiana University School of Medicine, Indianapolis, USA; 2 Department of Pediatrics, Division of Critical Care, Riley Hospital for Children, Indianapolis, USA; 3 Department of Pediatrics, Division of Critical Care, Indiana University School of Medicine, Indianapolis, USA

**Keywords:** cardiac arrhythmia, ventricular tachycardia (vt), electrolyte disturbances, global cerebral edema, diabetic ketoacidosis (dka)

## Abstract

Diabetic ketoacidosis (DKA) is known to cause total body potassium depletion, but during initial presentation, very few patients are hypokalemic, and even fewer patients experience clinical effects. As the correction of acidosis and insulin drive potassium intracellularly, measured serum potassium levels decrease and require repletion. This phenomenon is well described, and severe hypokalemia necessitates delaying insulin therapy. Less well described is the kaliuretic nature of treatments of cerebral edema. We present a case of an adolescent male with new-onset type 2 diabetes who presented in DKA with signs of cerebral edema, hyperosmolarity, and hypokalemia. As insulin and cerebral edema therapy were initiated, his hypokalemia worsened despite significant IV repletion, eventually leading to ventricular tachycardia and cardiac arrest. Over the following 36 hours, the patient received >590 milliequivalents (mEq) of potassium. He was discharged home 12 days after admission without sequelae of his cardiac arrest.

## Introduction

Diabetic ketoacidosis (DKA) is a metabolic state related to insulin deficiency characterized by an anion gap metabolic acidosis. Insulin deficiency results in the inability to utilize glucose, leading to the formation of ketones as an alternative energy source and a state of hyperglycemia. Hyperglycemia leads to an osmotic diuresis as the renal threshold to reabsorb glucose is surpassed, leading to profound dehydration and electrolyte loss [1]. According to the American Diabetes Association, the biochemical criteria for the diagnosis of DKA are hyperglycemia (blood glucose > 11 mmol/L or 200 mg/dL), venous pH < 7.3, or serum bicarbonate < 15, and ketonemia or moderate to large ketonuria [2]. Hyperglycemia results in the early manifestations of polyuria and polydipsia. Clinical signs include dehydration, tachycardia, Kussmaul breathing, nausea and vomiting, fruity breath, abdominal pain, and progressively deteriorating consciousness [2].

Potassium deficiency can be caused by any combination of osmotic diuresis, poor oral intake, and gastrointestinal losses. However, patients in DKA usually have a normal serum potassium level prior to the administration of insulin because insulin deficiency and acidosis both shift potassium extracellularly [3]. Serum hypokalemia usually occurs after the administration of insulin, as insulin itself and the resulting improvement in acidosis will both shift potassium intracellularly. The standard of care in the treatment of DKA is the inclusion of potassium in IV fluids during correction of hyperglycemia and to delay initiation of insulin until serum potassium is >2.5 mmol/L to avoid potentially fatal side effects of hypokalemia [4,5]. Hypokalemia is likely exacerbated in patients who require treatment for cerebral edema. Mannitol and hypertonic saline are known to result in hypokalemia due to osmotic diuresis. We report a case of an adolescent male with DKA and cerebral edema who suffered a ventricular arrhythmia and cardiac arrest as a result of refractory hypokalemia.

## Case presentation

A 13-year-old male with a history of obesity (admission weight: 99.4 kilograms) and fatty liver disease initially presented to his outpatient primary care physician with one week of fatigue and emesis. At that time, he was appearing well with a normal physical examination and normal vital signs. He was discharged home with ondansetron for symptom control from suspected viral gastroenteritis. Four days later, he presented to a local emergency department with the same symptoms as well as new drowsiness. He was found to be hyperglycemic with blood glucose > 500 mg/dL with positive serum ketones and a profound metabolic acidosis consistent with severe DKA (Table 1). He was given 3 liters of 0.9% NaCl as intravenous (IV) fluid boluses as well as an IV insulin bolus of 10 units. He was then started on an insulin infusion of 0.1 units/kilogram/hour (units/kg/hr). He was transported to the nearest pediatric intensive care unit (PICU) at a large tertiary referral center. He did not receive any potassium repletion prior to his arrival at our facility. During transport, he developed urinary incontinence and became more drowsy likely due to worsening cerebral edema, and was given 250 milliliters (mL) of 3% hypertonic saline. His potassium was <2.0 mmol/L at that time. His insulin infusion was stopped shortly after leaving the referring facility.

**Table 1:** Temporal relationship between laboratory values and potassium replacement. The patient's laboratory values from the presentation at the emergency room, through his cardiac arrest, and then on a full resolution of hypokalemia in relation to the total amount of potassium received throughout the 36 hours of his initial care. ^1 ^These are the point of care values and the first set of laboratory values in the ICU. ^2^ Potassium was reported as “slightly hemolyzed.” pCO2, partial pressure of carbon dioxide.

	10/14/20, 19:03	10/14/20, 20:35^1^	10/14/20, 21:35	10/15/20, 01:48	10/15/20, 06:37	10/15/20, 09:38	10/16/20, 08:19
Sodium (mmol/L)	131	139	142	143	149	147	163
Potassium (mmol/L)	2.7	<2.0	2.3^2^	2.1	2.2	1.9^2^	4.2
Chloride (mmol/L)	95		114	113	121	123	133
Bicarbonate (mmol/L)	5		4	4	7	6	14
Glucose (mg/dL)	914	570	496	532	485	581	436
Phosphorous (mg/dL)			1.1	1.6	1.6	1.1	1
Venous pH	6.99	7.01	6.96	7.05	7.13	7.08	7.23
Venous pCO2	16	<15	20				
Base excess (mmol/L)	−21		−26				
Insulin dose (u/kg/hr)			0.04	0.1		0.03	0.05
Cumulative potassium received (mEq)				84.4	124.4		697.3
Patient events					~2 hours before the arrest	Post-arrest	

On arrival to the PICU, the patient was somnolent but aroused to voice with a Glasgow Coma Scale (GCS) score of 12. He was tachycardic and tachypneic with Kussmaul breathing. He appeared moderately dehydrated but without evidence of shock. His laboratory evaluation was consistent with severe DKA with hypokalemia (Table 1). An EKG at that time was notable for sinus tachycardia with a prolonged corrected QT interval (616 per machine, 524 per hand calculation).

**Figure 1:**
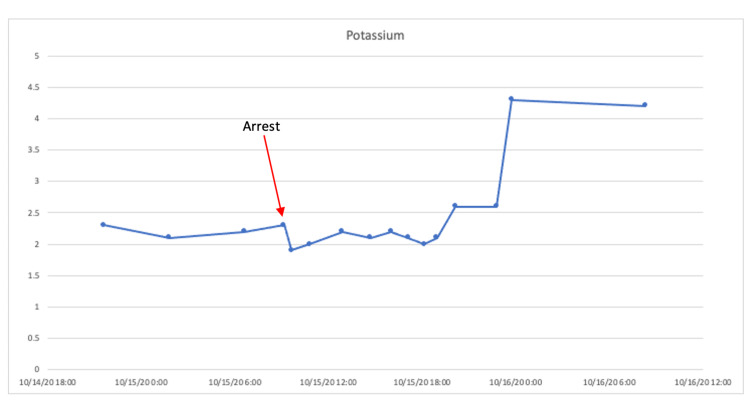
The patient’s serum potassium levels over time. Point of care values were removed for clarity of the figure. At the time of arrest, the patient’s serum potassium was 2.3 mmol/L.

The patient was started on the institutional DKA protocol with a two-bag fluid system. Prior to the initiation of an insulin infusion, he was given 40 mEq of IV potassium chloride (KCl) and 30 millimoles of potassium phosphate. An insulin infusion was started due to concern for cerebral edema; however, due to his hypokalemia, it was started at a lower rate (0.04 units/kg/hr) than what our protocol dictates (0.05-0.1 units/kg/hr). His serum potassium after initial repletion was 2.2 mmol/L. He was given another 40 mEq of IV KCl at that time. Approximately eight hours after admission, the patient had another episode of urinary incontinence and became less responsive (GCS score of 9). He was given an additional 250 mL of 3% hypertonic saline and 50 grams of mannitol. His mental status slightly improved (GCS score of 10). Two hours later, the patient went into stable ventricular tachycardia with a pulse. At that time, potassium on point of care electrolyte testing was found to be <2.0 mmol/L, and venous blood gas showed a pH of 6.94, bicarbonate of 10 mmol/L, and partial pressure of carbon dioxide (pCO2) of 48. Cardioversion was attempted twice, first with 100J then with 200J, but was unsuccessful in restoring a sinus rhythm. The patient ultimately deteriorated into a pulseless electrical activity cardiac arrest. After intubation and 12 minutes of cardiopulmonary resuscitation, the return of spontaneous circulation was achieved. He continued to have short runs of stable ventricular tachycardia, which was managed with an IV lidocaine bolus and a lidocaine infusion. His insulin infusion was turned off during the arrest and restarted following additional potassium repletion. For 24 hours after the code event, he continued to have refractory hypokalemia and required a total of 597 mEq of K as either KCl or potassium phosphate to correct to >3.0 mEq/L (Table 1 and Figure 1). Of note, plasma magnesium was noted to be normal at 2.0 mg/L throughout. After several days, he was extubated, recovered to a GCS score of 15, and ultimately transferred to the endocrinology service for further management of his diabetes. He was discharged home on long and short-acting insulin, and at an outpatient follow-up, he was ultimately diagnosed with type 2 diabetes, as autoantibodies were negative.

## Discussion

We present a case of severe hypokalemia resulting in cardiac arrest as the patient was being treated for DKA with cerebral edema. Approximately 40% of patients with new-onset type 1 diabetes who are admitted to the hospital will present in DKA, most commonly in children under the age of three years [6-8]. DKA does occur in type 2 diabetes, but is very uncommon, especially in children, such that it is difficult to find data on its true incidence. A 2004 study indicated 22% of patients admitted with DKA had type 2 diabetes; however, this study was done in adult patients [9]. The leading cause of death from DKA in children is cerebral edema, and once cerebral edema develops, the mortality rate is 20-25% [10].

The osmolar gradient caused by hyperglycemia in DKA results in a water shift from the intracellular to the extracellular space, leading to a reduction in cell volume. It has been theorized that when intravenous fluids and insulin are given to correct hyperglycemia, a rapid reduction in fluid osmolality results in a reversal of fluid shift intracellularly leading to the development of cerebral edema [11]. It has also been hypothesized that degree of dehydration rather than osmotic status may play a larger role in the development of cerebral edema due to a relative state of cerebral hypoperfusion before treatment, with subsequent vasogenic edema occurring during DKA treatment as a result of reperfusion of previously ischemic brain tissue [12]. Risk factors for the development of cerebral edema in DKA include younger age, a new diagnosis of diabetes, the severity of acidosis, lower pCO2 values, higher urea levels, administration of bicarbonate, larger volumes of fluid given over the first three to four hours, and administration of insulin within the first hour of fluid treatment [13,14]. Our patient met several of these criteria, with severe acidosis, pCO2 of 20 on admission, a large volume of fluid boluses prior to transfer, and administration of IV insulin within the first hour. Treatment should be initiated immediately with hypertonic saline or mannitol, although there remains debate about which agent is best [15]. While mannitol and hypertonic saline both treat cerebral edema, they also lead to increased kaliuresis.

Our patient’s severe hypokalemia presented a challenge in managing his progressive cerebral edema and profound acidosis [16]. Hypokalemia can cause a wide variety of cardiac arrhythmias, muscle necrosis/weakness, and respiratory depression [4]. Refractory or severe hypokalemia in DKA is rare, with only case reports in the literature of severe hypokalemia leading to life-threatening complications during treatment of DKA, one of which was related to inappropriate use of IV insulin [17]. In DKA, cardiac arrest in the setting of hypokalemia is exceedingly rare. Ventricular tachycardia and asystole have also been infrequently observed in DKA and adult patients have also shown Brugada-like syndrome and pseudo-myocardial infarction [18]. However, our patient had normal troponin levels.

In this patient, hypokalemia was closely monitored during IV repletion with adequate serum level maintenance. His immediate risk for mortality on presentation was felt to be his progressive cerebral edema and declining neurologic exam. Consequently, he was started on an insulin infusion at a low rate in an effort to improve his severe acidosis and drive for hypocapnia [19]. Worsening cerebral edema required treatment with hypertonic saline and mannitol, exacerbating his hypokalemia. We theorize that his potassium acutely dropped following its administration, which precipitated ventricular dysrhythmia and cardiac arrest. Our case highlights the importance of the presence of hypokalemia when managing cerebral edema in DKA.

## Conclusions

The treatment of refractory hypokalemia in the setting of cerebral edema in patients with DKA is difficult. Initiation of insulin, while imperative for treatment of DKA and associated cerebral edema, will worsen already existing hypokalemia. Additionally, treatments for cerebral edema including mannitol and hypertonic saline can also worsen hypokalemia. It is important for providers to recognize the effects of these treatments on serum potassium and how they may be complicated by refractory hypokalemia to closely follow serum potassium and replace it as necessary.
